# Habitats Are More Important Than Seasons in Shaping Soil Bacterial Communities on the Qinghai-Tibetan Plateau

**DOI:** 10.3390/microorganisms9081595

**Published:** 2021-07-27

**Authors:** Rui Wang, Miao Wang, Jing Wang, Yinghua Lin

**Affiliations:** 1 Institute of Wetland Research, Chinese Academy of Forestry, Beijing 100091, China; 17801238203@163.com (R.W.); wjgreeting@163.com (J.W.); 2 Party School of the Chengdu Committee of the Chinese Communist Party, Chengdu 610110, China; wm880215@hotmail.com

**Keywords:** Qinghai-Tibetan Plateau, permafrost habitats, bacterial communities, seasonal changes

## Abstract

Both habitats and seasons can determine the dynamics of microbial communities, but the relative importance of different habitats and seasonal changes in shaping the soil bacterial community structures on a small spatial scale in permafrost areas remains controversial. In this study, we explored the relative effect of four typical alpine meadow habitats (swamp wetland, swamp meadow, meadow and mature meadow) versus seasons on soil bacterial communities based on samples from the Qinghai-Tibetan Plateau in four months (March, May, July and September). The results showed that habitats, rather than seasons explained more variation of soil bacterial composition and structure. Environmental cofactors explained the greatest proportion of bacterial variation observed and can help elucidate the driving force of seasonal changes and habitats on bacterial communities. Soil temperature played the most important role in shaping bacterial beta diversities, followed by soil total nitrogen and pH. A group of microbial biomarkers, used as indicators of different months, were identified using random forest modeling, and for which relative abundance was shaped by different environmental factors. Furthermore, seasonality in bacterial co-occurrence patterns was observed. The data showed that co-occurrence relationships changed over months. The inter-taxa connections in May and July were more pronounced than that in March and September. *Bryobacter*, a genus of subgroup_22 affiliated to Acidobacteria, and *Pseudonocardia* belonging to Actinobacteria were observed as the keystone taxa in different months in the network. These results demonstrate that the bacterial community was clustered according to the seasonal mechanism, whereas the co-occurrence relationships changed over months, which indicated complex bacterial dynamics in a permafrost grassland on the eastern edge of Qinghai-Tibetan.

## 1. Introduction

Soil bacteria inhabiting permafrost regions can adapt to low-temperature conditions, and are a major contributor to maintaining ecosystem stability through litter decomposition and other basic ecological processes [1,2,3]. Both anthropogenic and natural factors affect the microbial communities in permafrost. Soil temperature, moisture, organic matter content and pH can influence the microbial community by directly regulating its physiological activities [4,5], while vegetation characteristics [5], spatiotemporal conversion [6], grazing intensity [7] and fertilizer management indirectly impact microbial communities by influencing nutrients in the soil [8]. Studies have shown that bacterial community structures vary among different habitats and months at small spatial scales due to changes in environmental conditions and ecological interactions [6,9]. Quantifying the contributions of habitats and seasonal shifts is critical to unravel the driving factors in microbial community dynamics.

Numerous studies in permafrost regions have shown that microbial community characteristics were regulated by habitats with different vegetation characteristics [10,11]. Vegetation types could largely influence the soil environment (e.g., pH, moisture and soil organic matter) via different litter input [9,12,13,14]. These variations subsequently lead to changes in soil carbon and nitrogen cycles, which eventually influence the rates of microbial metabolic processes [15,16]. Furthermore, plant morphology and vegetation cover can alter the transmission of UV-B to the soil surface, which may affect the soil microbial community [11,17]. However, other studies proved the contradictory pattern, indicating vegetation exerted little effect on soil microbial communities compared to other variables [18,19]. Thus, the influence of habitats with different vegetation characteristics on permafrost soil bacterial community structures on the Qinghai-Tibetan Plateau (QTP) remains an important controversial issue.

It is also clear that seasonal shifts serve as a determining effect in soil microbial dynamics, which derive from time-dependent factors such as soil moisture, temperature, nutrient level and vegetation biomass [20,21]. For decades, especially in agricultural and forest ecosystems, soil microbial communities have been observed to exhibit significant seasonal changes [5,22]. However, some conclusions from the same research area are contradictory [23]. Averill et al. [6] found equivalent community turnover in a soil microbial community over large intra-annual temporal differences and thousands of kilometers in space. In contrast, Zhang et al. [24] found that the time factor did not explain large-scale soil microbial diversity. Although former studies have provided critical information concerning temporal variability [25], the effect of seasonal shifts on the soil microbial community structure is still unclear in permafrost grassland ecosystems. In addition, there is little doubt that habitat effects and seasonal conversion may have a strong interaction in changing soil communities. The majority of previous studies usually used samples collected at a single point in time or sampling at the same location for multiple months. Therefore, the relative importance of different habitats versus months in shaping the dynamics of soil microbes remains to be discussed at small spatial scales.

The interactions between microorganisms are also important aspects of maintaining the diversity of microbial communities [26]. The co-occurrence patterns presented by the network could provide a reference in microbial ecology, regardless of the small amount of information reflecting the actual interaction [27]. Given the difficulty in obtaining microbial co-cultures, correlation-based network analysis serves as an alternative approach to infer microbial interactions and identify key species in ecological processes [28]. Whether microbial co-occurrence patterns vary with seasonal shifts remains unclear.

As the highest and largest plateau in the world, the altitude of QTP is 4000 m above sea level and contains the largest permafrost area at lower latitudes [29]. Alpine meadows are the dominant vegetation type of the QTP, accounting for about 50% of the available grassland area, which is fragile with poor anti-interference ability and sensitive to climate change [30]. At present, there have been substantial changes in vegetation structure along short geographical distances due to natural (climate, soil and vegetation) as well as anthropogenic (mass production and overgrazing) factors [31,32,33]. Consequently, we intended to elucidate whether habitats with different vegetation characteristics have dissimilar soil microbial community structures, and whether they follow the same seasonal change. This will aid in understanding the changes of microbial communities in responding to variations of grassland ecosystems over a period of time. In this study, an Illumina MiSeq was used to sequence 16S rRNA for detecting soil bacterial communities in the active-layer of permafrost from four typical alpine meadow habitats (wetland, swamp meadow, meadow, and mature meadow) on the eastern edge of Qinghai-Tibetan in different sampling months (March, May, July, September). By investigating the seasonal dynamics of soil bacterial communities under different habitats, we can better understand the influence of the physicochemical properties of soil, seasons and vegetation on the microbial composition and structure under natural conditions. The overall aims of this study were: (1) to analyze the soil bacterial composition and structure sampled from four typical alpine meadow habitats in different sampling month on the QTP; (2) to determine the contributions of environmental factors, seasonal shifts and habitats to differences in bacterial communities and investigate the relationship between environmental factors and bacterial communities; (3) to compare the co-occurrence patterns of bacterial communities over months and identify the potential keystone taxa in different seasons.

## 2. Materials and Methods

### 2.1. Study Area and Soil Sampling

This study was conducted at the Alpine Meadow and Wetland Ecosystem Research Station of Lanzhou University in Maqu County (Azi Branch Station, N33°40′, E101°52′), which is located in the east of the QTP at an altitude of 3350 m. The mean annual temperature is 1.3 °C (average temperature −11 °C in January and 11.8 °C in July), and the average yearly rainfall is 670 mm. The annual frostless season is less than 7 days, and the rainy season is concentrated during the short summer. The experimental site is mainly alpine meadow with many monocotyledons, including Poaceae and Cyperaceae [30]. The main soil type of the study area is meadow soil. The site is grazed only by livestock (e.g., yak (*Bos grunniens*) and Tibetan sheep (*Ovis aries*)), with a low degree of disturbance [34].

The classification of four typical alpine meadow habitats (100 m × 100 m) was based on the dominant vegetation and water gradient. Sampling sites were named according to the habitat of each as follows: swamp wetland, swamp meadow, meadow and mature meadow (Supplementary Figure S1; Table 1). Since the maximum distance between sampling points was less than 5 km, and all sites were within the same range of elevation (60 m), we did not consider geographic distance and precipitation differences among habitats. Soil samples were collected from different habitats at the same site on March, May, July and September in 2018. Five randomly selected sites (10 m × 10 m) were established along two diagonal lines in each habitat. Each site was considered a replicate, and all sites had the same slope and exposure. Five soil cores (20 cm depth) were randomly taken from each site using a soil sampler tube (over a 0.25 m^2^ area) and then mixed as one composite sample. Subsamples (approximately 50 g) were aseptically transferred into plastic bags and immediately sent to the laboratory on dry ice for soil bacterial community analysis. The remaining soil (approximately 200 g) was kept at 4 °C until the soil property measurements. After completing the above steps, we marked each sampling point so that we could accurately sample at the same site in the future.

### 2.2. Measurements of Environmental Factors

While collecting soil samples, five replicate temperature measurements were taken at a 10 cm depth using an infrared thermometer. Shoot biomass was clipped level to the ground from a quadrant (20 cm × 20 cm) at each of the five sampling sites within each habitat and then weighed after being oven-dried at 65 °C for 48 h. Soil subsamples were oven-dried at 105 °C until constant weight to calculate soil water content. The K_2_Cr_2_O_7_ method was used to determine the content of total organic carbon (TOC). Soil total nitrogen (TN) was determined using the semimicro Kjeldahl method. A pH meter (PHS-3C; Leici Instruments, Shanghai, China) was used to determine the soil pH value with a 1:2.5 ratio of soil to deionized water. For detailed descriptions of the soil chemical property measurement methods we used, see Bao et al. [35].

### 2.3. Total DNA Extraction and PCR Amplification

According to the standard kit protocol, total DNA was extracted from 0.5 g soil subsamples using a PowerSoil DNA extraction kit. The purity and quality of the genomic DNA were checked on 0.8% agarose gels. The primer sets 338F (ACTCCTACGGGAGGCAGCAG) and 806R (GGACTACHVGGGTWTCTAAT) were chosen to amplify the V3-V4 hypervariable regions of the bacterial 16S rRNA gene, given that this gene fragment provides sufficient resolution and has little bias for accurate classification of bacteria [36]. PCR was carried out on a Mastercycler Gradient (Eppendorf, Hamburg, Germany) using 25 µL reaction volumes containing 12.5 µL 2 × TaqPCR Master Mix, 3 µL BSA (2 ng/µL), 1 µL of each primer (5 µM), 2 µL template DNA, and 5.5 mL ddH_2_O. The cycling parameters were 95 °C for 5 min, followed by 32 cycles of 95 °C for 45 s, 55 °C for 50 s and 72 °C for 45 s with a final extension at 72 °C for 10 min. Three PCRs per sample were pooled to mitigate reaction-level PCR biases. The PCR products were purified using a QIA quick gel extraction kit (QIAGEN, Dusseldorf, Germany), and sequencing was performed on an Illumina MiSeq PE300 platform (Beijing Allwegene Technology Co., Ltd., Beijing, China).

### 2.4. Data Processing and Statistical Analyses

QIIME (version 1.17) was used to demultiplex and filter the original fastq files (http://qiime.org/scripts/pick_otus.html) [37]. Operational taxonomic units (OTUs) were clustered with a 97% similarity cutoff and rarefaction curves were generated. The rarefaction curves showed clear asymptotes, indicating that the bacterial communities were almost completely sampled (Supplementary Figure S2). The RDP classifier algorithm of the Silva (Silva 128) 16S rRNA database was used to classify each 16S rRNA gene sequence with an 80% confidence threshold. [38]. All samples were normalized at the same sequence depth (20,112). The relative abundance of each specific taxon was calculated as the percentage of sequences assigned to each taxon to the sequences in all samples. The species with a relative abundance of <1% in all samples were classified as others. The alpha diversity indices, including Sobs, Shannon, Chao 1 and pd indices, were calculated using “alpha_diversity. py”, while the beta diversity was calculated using “beta_diversity. py” in QIIME 1.8.0 software [39].

Boxplots showing the soil environmental variables in different months among four habitats were drawn using SPSS Statistics software (IBM Corporation, New York, NY, USA). A stack bar plot reflecting the distribution ratio of dominant bacterial phyla from different habitats and sampling months was performed in the R environment (package = “ggplot2”). The relative importance of different habitats and sampling months for explaining the variations in the soil environmental variables, the relative abundance of the dominant taxa and the alpha-diversity parameters were evaluated by one-way analysis of variance (ANOVA) in SPSS [24]. The explanation degree determined by habitats and seasons was represented by the R^2^-value. Permutational multivariate analysis of variance (PerMANOVA) tests followed by pairwise comparisons were performed on the weighted UniFrac distance in R (package = “vegan”) to test the significance of community distinctions among habitats and sampling months [40]. Variance partitioning analysis (VPA) (package = “vegan”) was used to evaluate the individual and interactive explanations of soil environmental factors, different habitats and sampling months on the differences in soil bacterial community structure. The influence of specific soil environmental factors on the dynamics of soil bacterial community structure was analyzed by distance-based redundancy analysis (db-RDA) (package = “vegan”) [41]. Classification random forest analysis (package = “randomForest”) was applied to select the most representative microbial biomarkers at the genus taxonomic level in all soil samples [24]. The number of decision trees was 500, and the accuracy of the model was verified by a 10-fold cross-validation method. The relationships of identified microbial biomarkers and soil environmental variables were estimated by Spearman correlations.

The co-occurrence pattern was determined by calculating the correlation between the top 50 genera, which were selected based on relative abundance. Genera were considered strongly correlated if the absolute value of Spearman’s correlation coefficient (r) was >0.6 and the *p*-value was <0.01 [26]. A correlation network was formed by the pairwise comparison of all significant correlations determined by genus abundance, in which each node represented a bacterial genus, and each edge indicated the significant correlation between nodes. In order to describe the topological structure of these networks, a set of measured values (including the number of nodes and edges, closeness centrality, betweenness centrality, average degree, degree centrality, clustering coefficient, and transitivity) were calculated in R, and Cytoscape was used to draw network diagrams [42].

## 3. Results

### 3.1. Distribution of Environmental Variables

All the environmental variables were found to be significantly affected by the different habitats and sampling months. Among the seven environment variables tested, TN, TOC and soil water content were largely influenced by habitat: 48.4% (*p* < 0.01), 57.4% (*p* < 0.01), and 81.8% (*p* < 0.01) of their respective variation were explained by habitat alone. In contrast, pH (R^2^ = 0.221, *p* < 0.05), C/N (R^2^ = 0.193, *p* < 0.01), shoot biomass (R^2^ = 0.777, *p* < 0.01), and soil temperature (R^2^ = 0.968, *p* < 0.01) were significantly influenced by the sampling month (Supplementary Figure S3). In September, the shoot biomass was significantly higher than in other months, yet the soil water content was lower. The soil temperature was lowest in March and reached a maximum in July. In addition, TN and TOC in the mature meadow were significantly lower than in the other meadow types, and the soil water content showed a downward trend across the four habitats (Supplementary Figure S3).

### 3.2. Soil Bacterial Diversities and Community Structure

**Figure 1:**
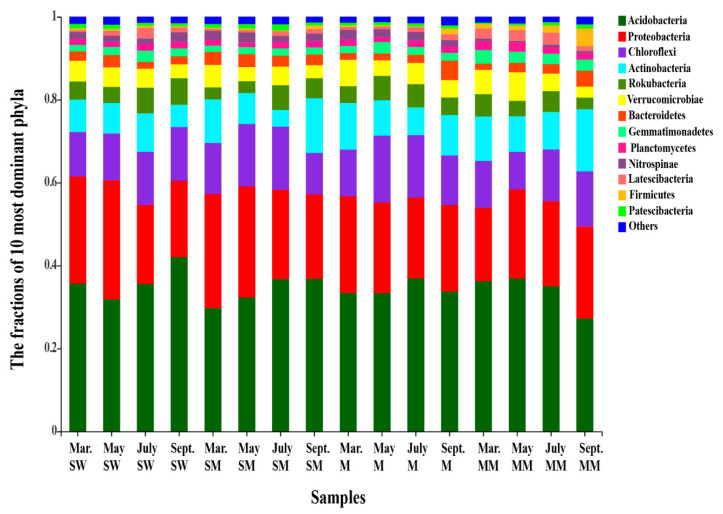
The fractions of the 10 most dominant phyla from different samples. The colors of the bars represent the bacterial phyla, and the length represents the relative abundance of the bacterial phyla in the corresponding sample. SW is swamp wetland, SM is swamp meadow, M is meadow, MM is mature meadow.

We obtained a total of 5,527,572 bacterial sequences. The average sequence length was 466 bp after removing low-quality sequences and chimeras. Each sample was sampled twice to keep the same sequencing depth (20,112 reads per sample) and the sample was clustered into 7556 OTUs at a 97% similarity level. A total of 50 bacterial phyla were detected in 80 soil samples. The dominant phyla with a relative abundance of more than 1% were Acidobacteria (34.59%), Proteobacteria (22.23%), Chloroflexi (12.55%), and Actinobacteria (9.02%). Circos showed contrasting community compositions over different habitats and months. In different habitats, Acidobacteria, Proteobacteria and Chloroflexi were the most abundant bacterial phyla, but the difference was not statistically significant among the four habitats (*p* > 0.05). Actinobacteria were more abundant in swamp meadows, meadows and mature meadows than in swamp wetlands (*p* < 0.05). Gemmatimonadetes and Planctomycetes were more abundant in mature meadows and swamp wetlands than in swamp meadows and meadows (*p* < 0.05). Comparatively, the samples of mature meadows had fewer Nitrospirae than the other three types (*p* < 0.05) (Figure 1). In different soil samples, Acidobacteria were always dominant, whereas Proteobacteria were more abundant in May than in other months (*p* < 0.05). Actinobacteria were more abundant in September and March than in May and July (*p* < 0.05). There were significant differences in the abundance of Rokubacteria, Verrucomicrobia and Latescibacteria in different month (*p* < 0.05) (Figure 1).

**Figure 2:**
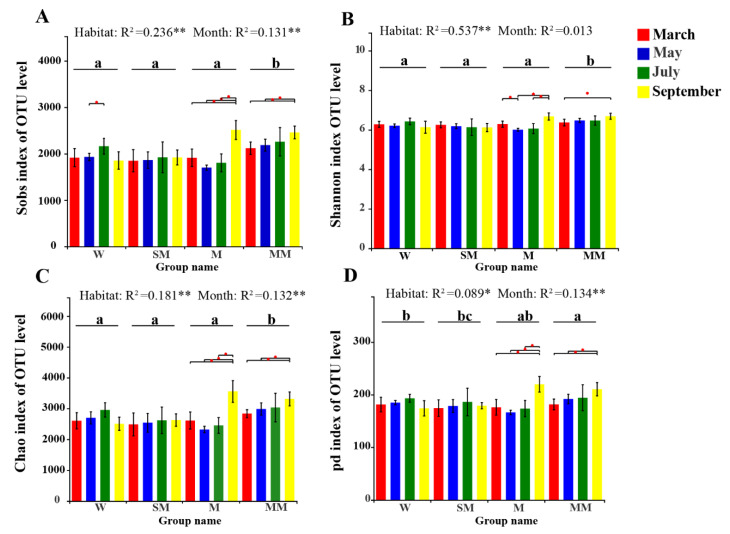
Estimated values of bacterial community Sobs index (**A**), Shannon index (**B**), Chao 1 index (**C**) and phylogenetic diversity (**D**). Significant differences are indicated by different letters. Significant differences among months in each habitat are marked by stars (** *p* < 0.01; * *p* < 0.05). The letters of “a”, “b” and “c” in the figure represent the post-hoc results following ANOVA analyses. Significant differences between different habitats are marked by different letters. SW is swamp wetland, SM is swamp meadow, M is meadow, MM is mature meadow.

There was no significant difference in the observed richness (Sobs), Shannon index or Chao1 estimator (Chao 1) among the different habitats, except in mature meadow (*p* < 0.05) (Figure 2). The samples from the mature meadows were found to have the highest levels of bacterial richness and diversity regardless of month. The phylogenetic diversity (PD) of swamp wetlands and swamp meadows was significantly different from that of mature meadows. In the meadow and mature meadow, the average values of alpha diversity among the different months appeared to be significantly different (Figure 2). In addition, the Sobs (R^2^ = 0.236, *p* < 0.01), Shannon index (R^2^ = 0.537, *p* < 0.01), and Chao 1 (R^2^ = 0.181, *p* < 0.01) were significantly influenced by the habitats. Nevertheless, 13.4% (*p* < 0.01) of the variation in PD was explained by sampling month alone, which was more than that explained by different habitats (Supplementary Table S1).

**Figure 3:**
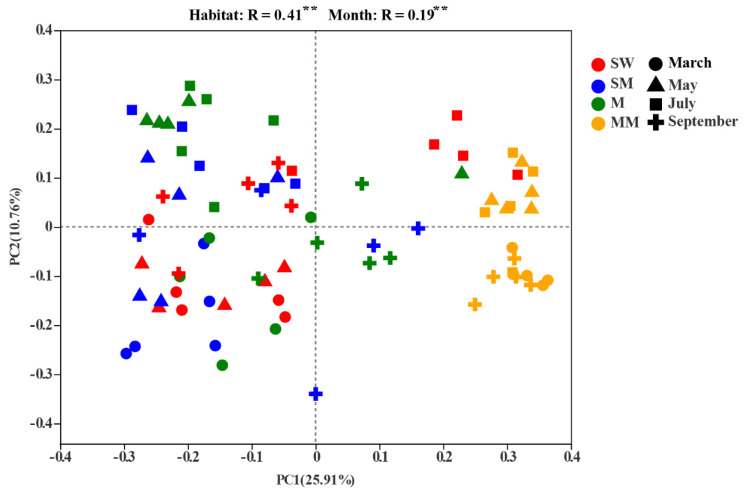
Principal-coordinate analysis (PCoA) plots of bacterial communities in all soil samples. The significant effect of month and habitat on microbial beta diversity was detected by PerMANOVA test. ** *p* < 0.01. SW is swamp wetland, SM is swamp meadow, M is meadow, MM is mature meadow.

The PCoA with the PerMANOVA test revealed that habitat and month had significant effects on bacterial community structure and that habitat (R = 0.41, *p* = 0.001) explained more bacterial community structure variation than month (R = 0.19, *p* = 0.001). The mature meadow was significantly separated from the other three habitats, but the others were not distinct from each other (Figure 3; Supplementary Table S3).

### 3.3. Effects of Environmental Factors on Bacterial Community Structure

**Figure 4:**
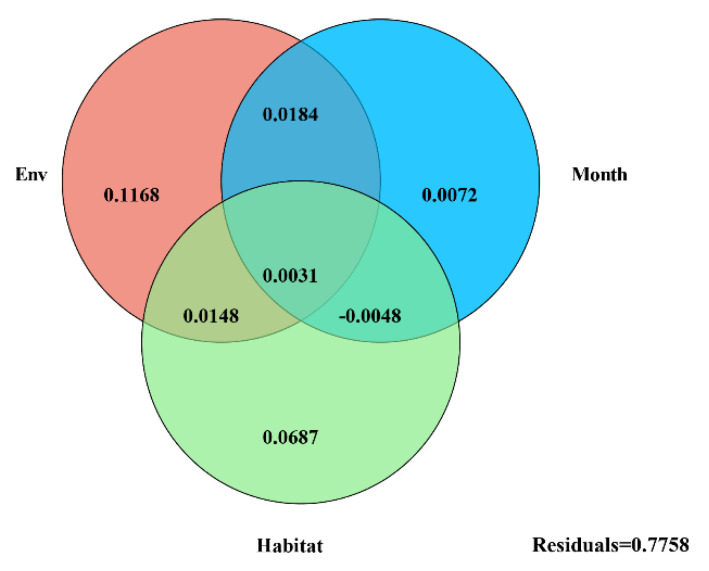
Variation partitioning analysis performed to quantify the contribution of environmental variables, sampling month and habitat to bacterial community variations.

**Figure 5:**
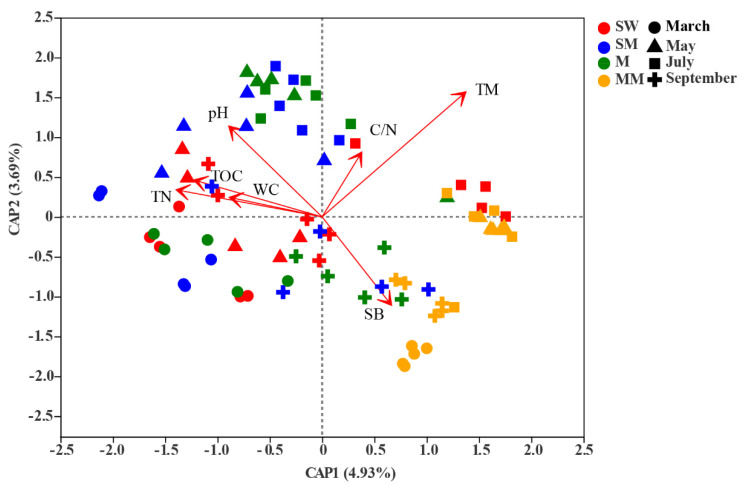
Distance-based redundancy analysis (db-RDA) of bacterial communities in all soil samples. The values of axes 1 and 2 are the percentages explained by the corresponding axis. TN is soil total nitrogen, WC is soil water content, SB is shoot biomass, TOC is soil total organic carbon, TM is soil temperature. SW is swamp wetland, SM is swamp meadow, M is meadow, MM is mature meadow.

According to VPA, environmental variables represented the largest fraction (15.31%) of variation in bacterial community composition followed by habitat (8.66%) and sampling month (2.87%). The three explanatory factors accounted for 0.31% of bacterial community variation, and 77.59% of the variance was unexplained, suggesting the complex assembly process of the bacterial community (Figure 4). The db-RDA indicated that the environmental variables of soil temperature (r^2^ = 0.588, *p* = 0.001), TN (r^2^ = 0.169, *p* = 0.003), pH (r^2^ = 0.161, *p* = 0.001), shoot biomass (r^2^ = 0.135, *p* = 0.004), TOC (r^2^ = 0.159, *p* = 0.004) and C/N (r^2^ = 0.100, *p* = 0.013) were the main factors that induced the shift in bacterial beta diversity. The soil water content (r^2^ = 0.066, *p* = 0.084) had no statistically significant effect on the bacterial community structure (Figure 5; Supplementary Table S2).

**Figure 6:**
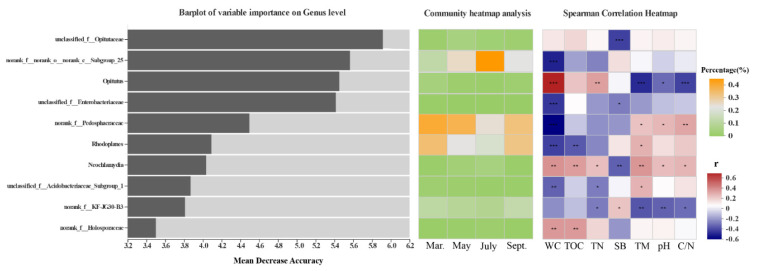
Top 10 identity biomarkers used for discriminating bacterial communities in different seasons (detected by random forest model). The assigned taxonomy of each taxon is displayed at the genus level. The bar plots on the left show the importance value of each biomarker estimated by the random forest model; the middle heatmap plots show the relative percentage of those biomarkers in different seasons; the right heatmaps show the Spearman correlations between the relative abundances of identity biomarkers and environmental variables. WC, soil water content; TOC, total organic carbon; TN, total nitrogen; SB, shoot biomass; TM, soil temperature; *** *p* < 0.001; ** *p* < 0.01; * *p* < 0.05.

Finally, a random forest model was used to distinguish microbial biomarkers that could discriminate the bacterial community in different seasons. Ranked by their importance value, the top 10 bacterial genera primarily belonged to *Opitutus*, *Rhodoplanes*, *Neochlamydia*, genera of Opitutaceae, Enterobacteriaceae and Pedosphaeraceae. These biomarkers were evenly distributed in the soil in different seasons. The relative abundances of *Opitutus*, *Neochlamydia* and unclassified genus-level lineage of the family Holosporaceae were strongly positively correlated with some of the environmental factors that were largely regulated by habitat (TN, TOC and water content), whereas *Rhodoplanes*, unclassified genus-level lineage of the family Acidobacteriaceae and Pedosphaeraceae were significantly negatively correlated with some of these factors. In contrast, these biomarkers had opposite reactions to pH, C/N, shoot biomass and soil temperature, which were largely influenced by the sampling season. Unclassified genus-level lineage of the family Opitutaceae, the biomarker with the highest score in importance measurement, had a significant negative correlation with shoot biomass (Figure 6).

### 3.4. Co-Occurrence Networks and Topological Properties of Bacteria in Different Months

**Figure 7:**
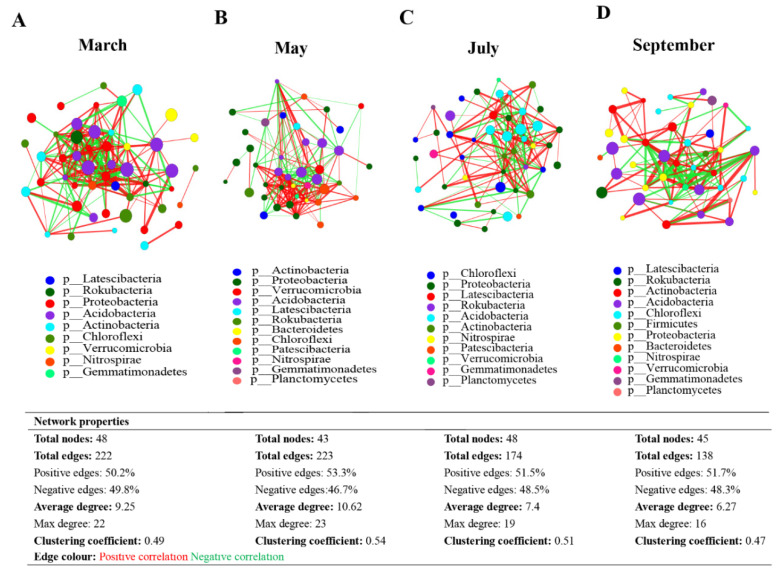
Co-occurrence networks and topological properties of bacteria from March (**A**), May (**B**), July (**C**) and September (**D**) soils. Nodes are colored according to microbial phylum, and the nodes with a larger size show the potential keystone genera (correlation coefficient ≥ 0.6, *p* < 0.01). Circle and square node shapes represent bacterial genus, respectively. Edges indicate correlations among nodes; the red and blue edges represent positive and negative correlations, respectively.

Network analyses were carried out to explore the co-occurrence among microbes and to identify potential keystone taxa. Based on the number of nodes and edges, average degrees and clustering coefficient, network complexities were comparable in different sampling months, and the complexity greatly increased in March and May. Generally, there were more positive correlations in each network, but the proportions of positive edges were higher in May than in other months. At the same time, the average degrees in the networks increased in the order of September, July, March and May. The representative biomarkers showed the highest clustering effect in different seasons. A genus of Subgroup_22 (degree = 22) affiliated with Solibacterales had the highest degree score in March. *Bryobacter* affiliated with Solibacteraceae exhibited high degrees in May (degree = 23) and July (degree = 19). *Pseudonocardia* affiliated with Actinobacteria was found to show the highest degree in September (degree = 16). Most of the nodes belonged to Acidobacteria, Proteobacteria, Rokubacteria and Chloroflexi in all networks, which implied their important role in the studied ecosystem (Figure 7).

## 4. Discussion

### 4.1. The Effect of Habitats Is Stronger Than Seasonal Changes on the Distribution of the Soil Bacterial Community

Previous studies on the temporal variation of soil microbial community in local areas showed that their structure had great seasonal variability [43,44]. The study of soil bacteria at different sites at the same sampling time also confirmed the important role of habitat in shaping bacterial community structure. However, there are few studies on soil microbial ecology during multiple sampling seasons in the same site. In this study, we investigated the patterns of soil bacterial communities from different habitats and sampling months to evaluate the independent effects of season and habitat on microbial community variations. Our results provide solid empirical evidence that habitats with different vegetation communities are more important than seasons for predicting the variation that characterizes the bacterial structure. Crucially, the results showed that season and habitat shape the structure of the soil bacterial community by adjusting relevant environmental factors.

Habitats have stronger influences in terms of changes in bacterial community composition, alpha diversity and beta diversity than does seasonal variability when assessed at a small spatial scale. Since microbial communities can differ at the scale of meters or centimeters [45], it is understandable that sampling month is not as important as sampling space, even at a small spatial scale [46,47]. However, in a study on the relationship between residual DNA and soil microbial communities on the relative hillsides of Colorado, USA, it was found that removing residual DNA from soil led to greater temporal changes [22]. That is, the time signal may be masked by residual DNA (DNA released from dead microorganisms) in the data. In our study, the investigation of the total DNA of bacteria did not consider the influence of residual DNA, which may underestimate the true extent of seasonal variation in the bacterial community. More than 77.6% of the variations in bacterial community dynamics were not explained by month, habitat or environmental variables in our study. The possible reason for this result may be the existence of other unmeasured environmental factors, which vary with habitat and season [25,48], including competition, symbiosis, predation and other biological interactions among microbial taxa microbial taxa [49,50] and ecological processes such as dormancy and persistence traits of microbial communities [6].

### 4.2. Environmental Variables Help Understand the Driving Effect of Habitats and Seasonal Shifts on Bacterial Communities

Different habitats affect bacterial communities by regulating related environmental factors. Habitats had a greater impact on TN (R^2^ = 0.484, *p* < 0.01), TOC (R^2^ = 0.574, *p* < 0.01) and soil water content (R^2^ = 0.818, *p* < 0.01). This made Actinobacteria and Nitrospirae differ significantly in various habitats (*p* < 0.05), which were more moisture-sensitive than representatives of other phyla [33,42]. Soil water content also has important effects on oxygen availability, and excessive water affects the aeration of the soil; thus, the environment becomes deoxygenated [51]. This process may explain why some anaerobic bacteria (i.e., *SM1A02* affiliated with Planctomycetes) increased significantly with changes in habitat (*p* < 0.05) [52,53]. The quality and quantity of litter and root exudates are often different between different plant communities, resulting in differences in soil nutrient contents (TN and TOC), and thus affect soil bacterial community structure [52,54]. The taxa within Proteobacteria can grow rapidly in nutrient-rich environments. Therefore, their relative abundance decreases as nutrient levels decline [55], whereas some genera affiliated with Actinobacteria, such as *Gaiella*, *Acidothermus* and *Conexibacter* whose relative abundance ranked within top 10 among the genera belonging to Actinobacteria, are well-adapted to oligotrophic environments, resulting in the enrichment of relative abundance as nutrients decrease [56]. Thus, the ratio of Actinobacteria to Proteobacteria gradually increased as the habitats varied from swamp wetland to mature meadow in this study, and the observed shifts were consistent with decreased soil nutrient status (i.e., carbon and nitrogen content). Zhang et al. [33] used the increase in the Actinobacteria to Proteobacteria ratio as a signal of permafrost degradation on the QTP, and these results were supported by this study, suggesting the necessity of more attention in this area.

In the same habitat, there were significant differences in bacterial communities in different sampling months. The bacterial communities attained higher diversity in July and September. Microbial taxonomic richness is known to reflect metabolic diversity [57]. Thus, the greater species diversity in this period probably derived from high enzyme activity promoted by favorable temperatures, or the higher biomass and diversity of shoot and root exudates in a given month [58]. Seasonal shift has greater influence than habitat only in PD α diversity index differences, which proved the important role of different months in shaping bacterial phylogenetic diversity. In the formation of bacterial community structure, soil temperature was the major influence and was largely affected by the sampling month (r^2^ = 0.588, *p* = 0.001). Changes in soil temperature directly affect microbial metabolic function and soil organic matter cycling in ecosystems [59], and previous studies proved that soil temperature is an important factor affecting soil microbial community structure [10,60,61]. However, this experiment was carried out in a permafrost area, and the increase in temperature would lead to a coordinated change in soil moisture content. The difference in hydrothermal conditions may have a very large impact [62,63]. Shoot biomass (r^2^ = 0.135, *p* = 0.004) and pH (r^2^ = 0.161, *p* = 0.001) had significant impacts on bacterial community structure, which was mainly regulated by month. Soil pH is known as a strong driver shaping the bacterial community in various soil ecosystems [47,64,65]. It has been proven that changes in pH on a small scale are difficult to detect; thus, the variation in pH in different habitats was smaller than that in sampling months [66,67]. Shoot biomass is one of the most important factors affecting the quality of nutrients returned by plants and the rate of photosynthesis [53]. From March to September, the biomass of aboveground vegetation increased gradually, which directly increased the impact on the underground bacterial community.

Many previous studies that focused on temporal variation in bacterial composition have implicated that it is often induced by the availability of nutrients, temperature, and moisture [68,69]. However, not all taxa within the bacterial community are equally sensitive to temporal changes in the environment [44]. In our study, we detected certain taxa that contributed to the discrimination of bacterial communities in different months. Their relative abundances did not occupy dominant positions in the whole community, but they did play important roles in indicating the seasonal variation of the community, suggesting that some nondominant taxa play a vital role in the ecological process. The complex and staggered relationship between these important phyla of bacteria and environmental factors made the ecological process more undefined. *Neochlamydia* belonging to Chlamydia is an important biomarker in terms of indicating the seasonal changes in the bacterial community. This biomarker cannot synthesize bioenergy substances (ATP) and is therefore completely dependent on the supply of the infected host cell [70]. *Neochlamydia* exhibited significant correlation with all environmental factors in our study, and the possible reason for this result was that their hosts (plants or other organisms) were strongly affected by the monthly variation; this needs further study. Another interesting result was that most biomarkers showed opposite responses to TN, TOC, soil water content (soil environmental variables greatly affected by habitat), pH, C/N, shoot biomass, and soil temperature (largely influenced by seasonal shifts), indicating that habitats and seasonal shifts may have antagonistic effects on the shaping of bacterial communities. The results of VPA also verified this conclusion.

### 4.3. Bacterial Network Interactions in Different Sampling Months

Co-occurrence networks showed different patterns in different months and they could partially reveal complex interactions within a bacterial community [27,71]. The positive correlations between bacteria were greater than the negative correlations in all soil samples, indicating that different bacteria responded to the environmental factors in a more similar way than a competitive way. The most abundant species of networks were Acidobacteria, Proteobacteria, and Chloroflexi, suggesting that these “generalists” adapt to various environments and play a key role in ecological processes [28,46,72]. A genus of Subgroup_22 and *Bryobacter* affiliated with Acidobacteria showed the highest clustering effects in different months. Several studies have confirmed that Acidobacteria is among the most dominant phyla in many soil ecosystems due to its ability to break down, utilize, and biosynthesize diverse structural and storage polysaccharides in various environments [73,74]. The genus *Bryobacter* accommodates acidotolerant, strictly aerobic, slow-growing chemoorganotrophic bacteria, which inhabit acidic wetlands and soils in our study [75]. In September, *Pseudonocardia*, belonging to a kind of rare actinomycetes, had the highest degree of connection with other bacteria. *Pseudonocardia* is mainly related to cellulose degradation and antibiotic synthesis which can produce some important enzymes and vitamins. They are also important in plant-associated microbial communities and referred to as “free-living” which allows these organisms to require less energy and food for survival [76,77]. This may be one of the potential reasons for the change in TN in different habitats in September. May and July showed more clustering coefficients than in other months, which may be related to the environmental conditions. In March, the temperature was low, and plants had just started growing. The bacterial community broke dormancy and became more active at this time. The enzyme activity was still very low, so there were fewer relationships between bacteria. In September, the biomass of plants reached a maximum and the ecosystem tended to be stable. The interaction between bacteria was reduced, so a relatively scattered distribution pattern formed. In May and July, the temperature was suitable, the amount of rain was sufficient, and the enzyme activity was highest. Plants grow rapidly to drastically change soil nutrient conditions, and members of the bacterial community must quickly adapt to the environmental changes.

## 5. Conclusions

Our study confirmed that the soil bacterial community changed with different seasons and habitats. The effects of four representative habitats on bacterial structure on a small scale were much greater than those of seasons. The correlation analysis of the bacterial community and environmental factors provided further evidence that habitats and seasonal shifts affected the soil bacterial community structure by changing the soil physicochemical and plant properties. Some keystone species (a genus of Subgroup_22, *Pseudonocardia* and *Bryobacter*) were closely related to other bacterial groups and played important roles in ecological processes. These results indicate that, to a certain extent, the data obtained from the snapshot study can roughly predict the distribution pattern and small-scale seasonal changes of bacteria in different habitats, which can be partly explained by environmental factors. However, caution should be taken when interpreting our results, given that only four months of the growing season were included in our study. The influence of aboveground vegetation on underground microorganisms is greatly weakened in winter when it is cold and there is little light, which causes obvious environmental differences in natural ecosystems. The limited time points used in this study might underestimate the true bacterial seasonal variation; hence, more winter month points should be included when designing future studies. In summary, the study of soil bacterial ecology at both habitat and season levels is most likely to provide a more comprehensive understanding of the key factors regulating the biodiversity of permafrost grassland ecosystems on the QTP.

## Figures and Tables

**Table 1:** Characteristics of the four typical alpine meadow habitats.

Habitats	Altitude	Latitude & Longitude	Dominant Plant	Description (Ma et al., 2011)
SW	3512 m	33°39′ N 101°32′ E	*Festuca sinensis* Keng ex S. L. Lu *Kobresia royleana* (Nees) Bocklr. *Potentilla anserina* L.	The dominant species are typical wetland plants grazed by yak and Tibetan sheep.
SM	3522 m	33°39′ N 101°52′ E	*Ranunculus japonicus* Thunb. *Carex tristachya* Thunb. *Leontopodium nanum* (Hook. f. et Thoms.) Hand.-Mazz. *Festuca sinensis* Keng ex S. L. Lu	The habitat is drier than SW. Water is distributed on the soil surface in some parts of this habitat only in the rainy season. The swamp meadow has been lightly grazed by yak and Tibetan sheep.
M	3540 m	33°39′ N 101°53′ E	*Pleurospermum camtschaticum* Hoffm. *Carex tristachya* Thunb. *Sanguisorba filiformis* (Hook. f.) Hand.-Mazz. *Festuca sinensis* Keng ex S. L. Lu	The habitat is drier than SM, without water on the soil surface at any time. It has already become meadow with some drought-tolerant vegetation.
MM	3572 m	34°40′ N 102°52′ E	*Artemisia mongolica* (Fisch. ex Bess.) Nakai *Elymus nutans* Griseb. *Anemone obtusiloba* D. Don *Pedicularis szetschuanica* Maxim.	It is a mature alpine meadow and soil moisture content is the lowest among four habitats. The mature meadow is under low disturbance of grazing.

SW is swamp wetland, SM is swamp meadow, M is meadow, and MM is mature meadow.

## Data Availability

All data and material produced from this study are provided in this manuscript. The datasets presented in this study can be found in online repositories. The names of the repository and accession number can be found below: https://www.ncbi.nlm.nih.gov/, PRJNA669123.

## Supplementary Materials

The following are available online at https://www.mdpi.com/article/10.3390/microorganisms9081595/s1, Figure S1: Characteristics of study sites in different habitats and sampling months, Figure S2: Rarefaction curve of coverage index on OTU level of all soil samples, Figure S3: Boxplots showing the soil environmental variables in different months among the four habitats, Table S1: Alpha diversity index in different habitats and months soil samples, Table S2: Relevant data sheets of db-RDA in different months, Table S3: R^2^ value of pairwise comparisons using PERMANOVA based on weighted UniFrac distances.
