# Supplementary material for: Habitats Are More Important Than Seasons in Shaping Soil Bacterial Communities on the Qinghai-Tibetan Plateau
Source: Microorganisms. 2021 Jul 27;9(8):1595. doi: 10.3390/microorganisms9081595 (PMC8400953; doi:10.3390/microorganisms9081595)
Supplement: Supplementary file 1 [file microorganisms-09-01595-s001.zip › microorganisms-1271401-supplementary.pdf]

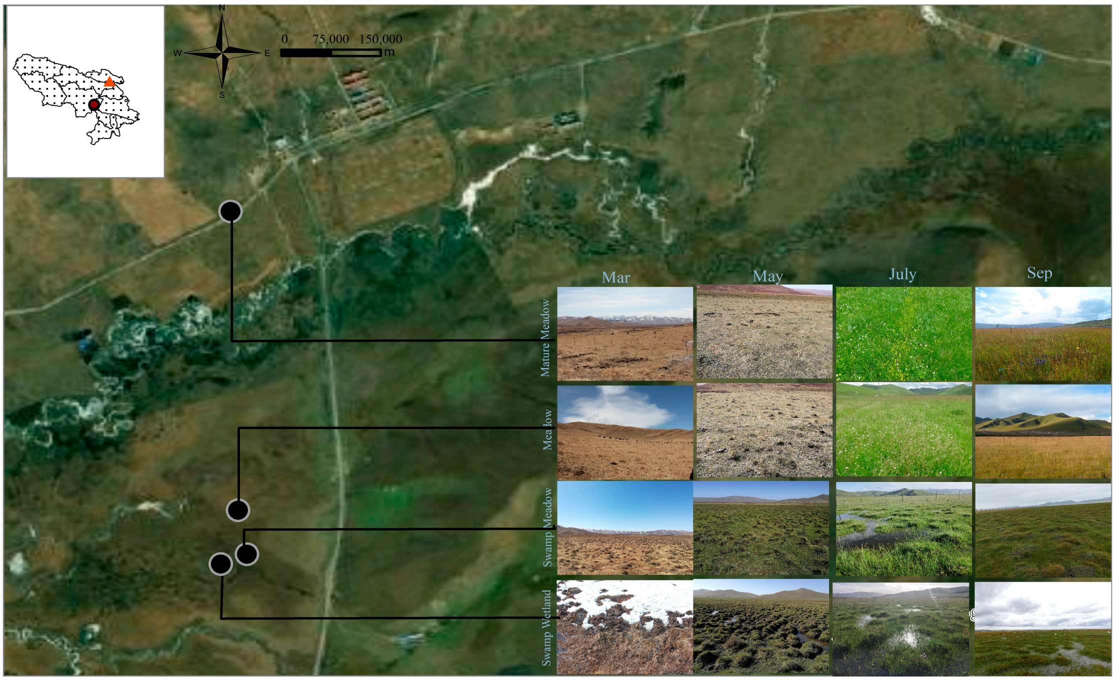

Supplementary Figure S1 | Characteristics of study sites in different habitats and sampling months. The dark circle and red triangle on the upper left map panel respectively represents sampling site and the location of county government.

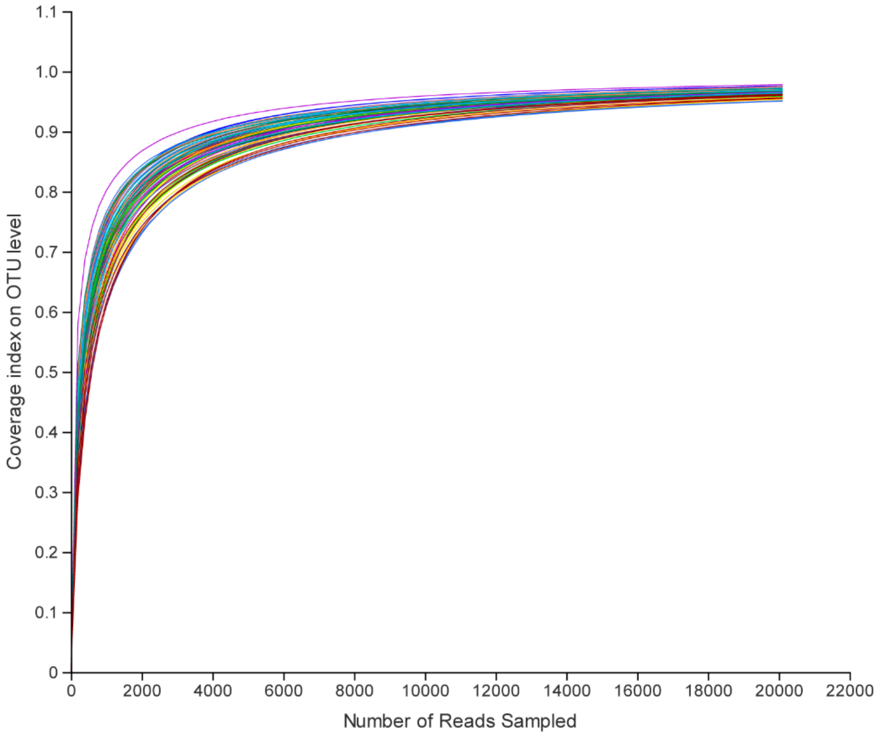

Supplementary Figure S2 | Rarefaction curve of coverage index on OTU level of all soil samples.

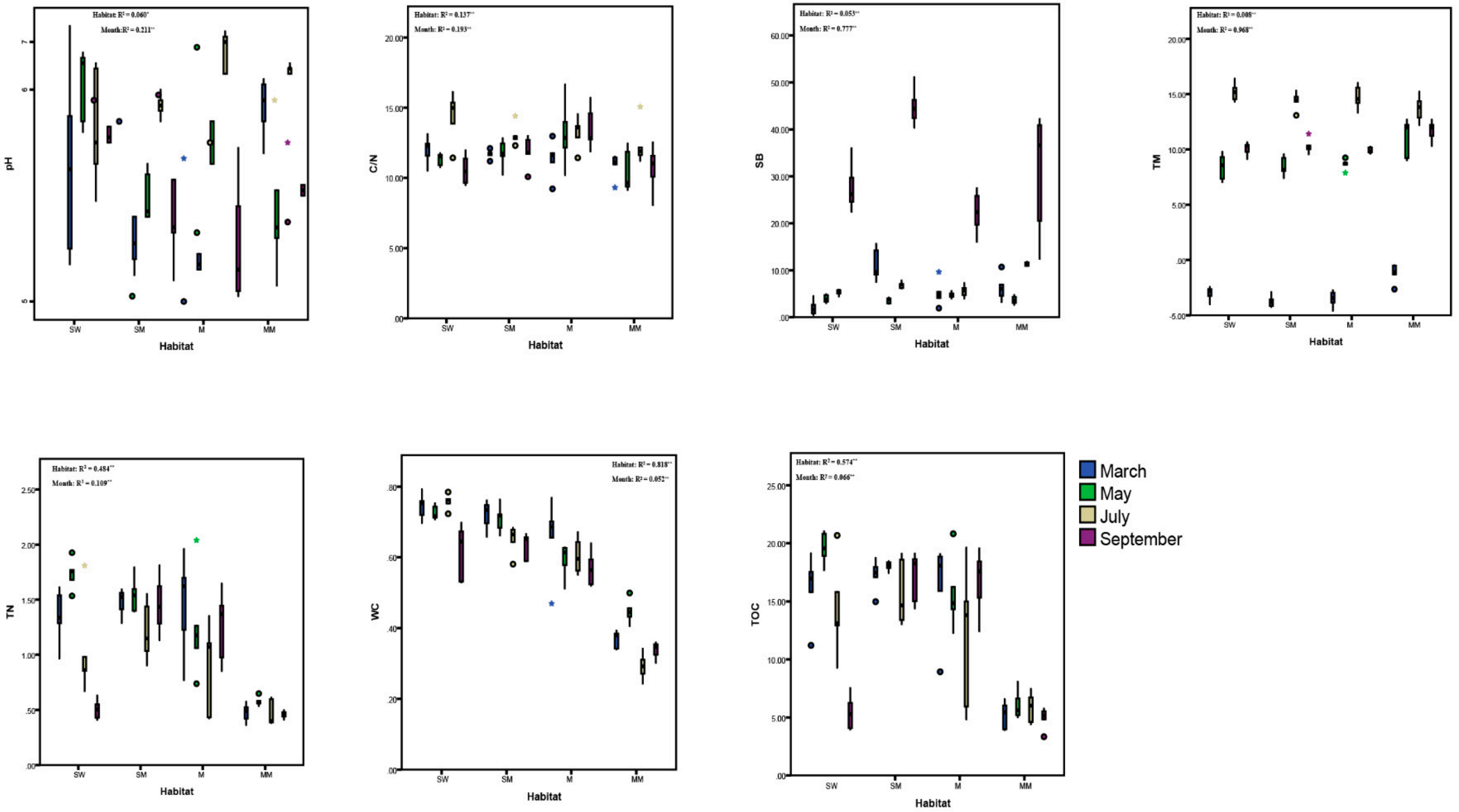

Supplementary Figure S3 | Boxplots showing the soil environmental variables in different months among the four habitats. The significant effect of months and habitat on each environmental variable was detected by one-way ANOVA. WC, soil water content; TOC, total organic carbon; TN, total nitrogen; SB, shoot biomass; TM, soil temperature based on the sampling days; ** $P < 0.01$; * $P < 0.05$. SW is swamp wetland, SM is swamp meadow, M is meadow, MM is mature meadow.

Supplementary Table S1 Alpha diversity index in different habitats and months soil samples. SW is swamp wetland, SM is swamp meadow, M is meadow, MM is mature meadow.

Habitat	Month	Read number	Sobs	Shannon	Chao	PD
W	March	20112	1915.6±195.17	6.277±0.150	2602.0±264.31	181.29±13.956
	May	20112	1930.8±78.79	6.207±0.094	2696.5±197.89	185.00±3.990
	July	20112	2160.8±169.32	6.423±0.169	2955.8±231.00	193.15±7.557
	September	20112	1853.2±187.65	6.132±0.303	2503.8±214.99	174.16±14.479
SM	March	20112	1849.0±239.43	6.252±0.148	2482.3±371.61	174.58±15.843
	May	20112	1861.2±177.86	6.186±0.123	2537.6±304.18	178.53±12.337
	July	20112	1922.2±332.10	6.132±0.420	2616.9±431.46	186.23±26.322
	September	20112	1919.4±160.83	6.120±0.208	2624.7±202.86	179.10±5.953
M	March	20112	1913.6±189.07	6.288±0.153	2611.2±276.96	176.06±14.839
	May	20112	1699.2±55.28	6.005±0.072	2317.0±105.02	166.27±4.413
	July	20112	1803.2±193.10	6.061±0.253	2451.3±255.94	173.61±15.430
	September	20112	2512.0±204.68	6.675±0.181	3553.5±352.57	219.83±14.803
MM	March	20112	2118.6±133.98	6.369±0.171	2832.5±128.74	181.64±10.185
	May	20112	2184.8±127.88	6.473±0.107	2984.4±197.44	191.82±9.107
	July	20112	2258.0±305.04	6.468±0.235	3031.4±464.09	194.34±24.663
	September	20112	2458.2±136.42	6.689±0.155	3310.4±225.24	210.46±12.701

Supplementary Table S2 Relevant data sheets of db-RDA in different months. r^2 is the determination coefficient, P is the significance test value.

Environment factors	r^2	P-values
TM	0.5883	0.001
TN	0.1685	0.003
pH	0.1606	0.001
SB	0.135	0.004
TOC	0.1319	0.004
C/N	0.0999	0.013
WC	0.0657	0.084

WC is soil water content, TOC is total organic carbon, TN is total nitrogen, SB is shoot biomass, TM is soil temperature.

Supplementary Table S3 R^2 value of pairwise comparisons using PERMANOVA based on weighted UniFrac distances

	SW	SM	M
**SM**	0.03	-	-
**M**	0.04	0.04	-
**MM**	**0.22**	**0.27**	**0.26**

	March	May	July
**May**	**0.09**	-	-
**July**	**0.14**	**0.04**	-
**September**	**0.07**	**0.07**	**0.09**

The R^2 value indicates the degree of explanation of the grouping factor to the sample difference. Bold font indicates $P < 0.05$, indicating that the reliability of the test is high. SW is swamp wetland, SM is swamp meadow, M is meadow, MM is mature meadow.
